# Supplementary material for: The effects of genital myiasis on the diversity of the vaginal microbiota in female Bactrian camels
Source: BMC Vet Res. 2022 Mar 5;18:87. doi: 10.1186/s12917-022-03189-5 (PMC8897907; doi:10.1186/s12917-022-03189-5)
Supplement: Supplementary file 5 — Additional file 5. [file 12917_2022_3189_MOESM5_ESM.zip › MPL201709200_16s_yy/Treat1/css/js_css/jsPDF-master.zip!/jsPDF-master/docs/jsPDF.html]

jsPDF - Documentation


## Home

### Classes

- jsPDF

### Global

- addFont
- addHTML
- addMetadata
- addPage
- autoPrint
- CapJoinStyles
- circle
- ellipse
- getFontList
- lines
- lstext
- output
- rect
- roundedRect
- save
- setDisplayMode
- setDrawColor
- setFillColor
- setFont
- setFontSize
- setFontStyle
- setLineCap
- setLineJoin
- setLineWidth
- setPage
- setProperties
- setTextColor
- text
- triangle

# jsPDF

## jsPDF

#### new jsPDF(orientation, unit, format) → {jsPDF}

Source:
:   - jspdf.js, line 47

Creates new jsPDF document object instance.

##### Parameters:

| Name | Type | Description |
| --- | --- | --- |
| `orientation` |  | One of "portrait" or "landscape" (or shortcuts "p" (Default), "l") |
| `unit` |  | Measurement unit to be used when coordinates are specified. One of "pt" (points), "mm" (Default), "cm", "in" |
| `format` |  | One of 'pageFormats' as shown below, default: a4 |

##### Returns:

Type
:   jsPDF

### Members

#### (static) API

Source:
:   - jspdf.js, line 2217

jsPDF.API is a STATIC property of jsPDF class.
jsPDF.API is an object you can add methods and properties to.
The methods / properties you add will show up in new jsPDF objects.

One property is prepopulated. It is the 'events' Object. Plugin authors can add topics,
callbacks to this object. These will be reassigned to all new instances of jsPDF.
Examples:
jsPDF.API.events['initialized'] = function(){ 'this' is API object }
jsPDF.API.events['addFont'] = function(added\_font\_object){ 'this' is API object }

##### Example

```
jsPDF.API.mymethod = function(){
  // 'this' will be ref to internal API object. see jsPDF source
  // , so you can refer to built-in methods like so:
  //     this.line(....)
  //     this.text(....)
}
var pdfdoc = new jsPDF()
pdfdoc.mymethod() // <- !!!!!!
```

  

Documentation generated by JSDoc 3.4.2 on Sun Oct 09 2016 11:08:27 GMT+0100 (BST) using the docdash theme.
